# Solubility and bacterial sealing ability of MTA and root-end filling materials

**DOI:** 10.1590/1678-775720150437

**Published:** 2016

**Authors:** Camila Galletti ESPIR, Juliane Maria GUERREIRO-TANOMARU, Rubens SPIN-NETO, Gisselle Moraima CHÁVEZ-ANDRADE, Fabio Luiz Camargo Villela BERBERT, Mario TANOMARU-FILHO

**Affiliations:** 1 - Universidade Estadual Paulista, Faculdade de Odontologia de Araraquara, Departamento de Odontologia Restauradora, Araraquara, São Paulo, SP, Brasil.; 2 - Department of Dentistry, Oral Radiology, Aarhus University, Aarhus, Denmark.

**Keywords:** Endodontics, Calcium silicate, Retrograde obturation, Solubility, Dental leakage

## Abstract

**Objective:**

To evaluate solubility and sealing ability of Mineral Trioxide Aggregate (MTA) and root-end filling materials.

**Material and Methods:**

The materials evaluated were: MTA, Calcium Silicate Cement with zirconium oxide (CSC/ZrO_2_), and zinc oxide/eugenol (ZOE). Solubility test was performed according to ANSI/ADA. The difference between initial and final mass of the materials was analyzed after immersion in distilled water for 7 and 30 days. Retrograde cavities in human teeth with single straight root canal were performed by using ultrasonic tip CVD 9.5107-8. The cavities were filled with the evaluated materials to evaluate sealing ability using the bacterial leakage test with *Enterococcus faecalis*. Bacterial leakage was evaluated every 24 hours for six weeks observing the turbidity of Brain Heart infusion (BHI) medium in contact with root apex. Data were submitted to ANOVA followed by Tukey tests (solubility), and Kruskal-Wallis and Dunn tests (sealing ability) at a 5% significance level.

**Results:**

For the 7-day period, ZOE presented highest solubility when compared with the other groups (p<0.05). For the 30-day period, no difference was observed among the materials. Lower bacterial leakage was observed for MTA and CSC/ZrO_2_, and both presented better results than ZOE (p<0.05).

**Conclusion:**

MTA and CSC/ZrO_2_ presented better bacterial sealing capacity, which may be related to lower initial solubility observed for these materials in relation to ZOE.

## INTRODUCTION

Dimensional stability, insolubility in tissues fluids, and sealing ability are ideal properties for a root-end filling material^23^. Solubility is evaluated based on material mass loss after immersion in water for 24 hours. MTA is a calcium silicate-based cement (CSC) and it has been one of the most studied repair materials, showing excellent biological and satisfactory physicochemical properties^6^. In comparison with Portland cement, MTA shows higher solubility^17^. Although this analysis is performed using a 24-hour period for evaluation, longer periods have been used and may be important to understand materials properties^14^.

Both the dimensional change and solubility may be directly related to material sealing ability. Several studies evaluate the bacterial leakage in different methodologies^20^ ^,^ ^22^ ^-^ ^24^. These studies suggest that proper dimensional stability and insolubility contribute to a lower degree of bacterial and fluid leakage.

Some physicochemical properties of MTA may be improved by replacing bismuth oxide (Bi_2_O_3_) used as radiopacifier^5^ ^,^ ^13^. The addition of 30% zirconium oxide to Portland cement promotes better properties such as mechanical strength, calcium release, increase in pH, and better biologic response and solubility than MTA^27^. Zirconium oxide (ZrO_2_) has been proposed as radiopacifier for cements based on calcium silicate and Portland cement such as tricalcium silicate and Biodentine^14^ ^,^ ^27^.

The aim of this study was to evaluate the solubility and sealing ability of MTA, calcium silicate cement with zirconium oxide (CSC/ZrO_2_), and zinc oxide/eugenol (ZOE) cement. The null hypothesis is that the materials are similar and the addition of zirconium oxide does not interfere in MTA properties.

## MATERIAL AND METHODS

For this experiment, the evaluated materials were: MTA, CSC/ZrO_2_, and ZOE. MTA (Angelus, Londrina, PR, Brazil) was manipulated in the powder/liquid proportion of 1 g/330 µL. Portland cement (Votorantim, São Paulo, SP, Brazil) with 30% ZrO_2_ (Sigma, St. Louis, MO, USA) was used with ratio 1 g powder to 320 µL distilled water. Zinc oxide and eugenol cement (S.S.White Art. Dent. Ltda., Rio de Janeiro, RJ, Brazil) were used with ratio 1 g zinc oxide to 0.2 mL eugenol. Samples were maintained at 37°C and 100% humidity for 24 hours before performing the tests.

### Solubility

This test was performed according to ANSI/ADA specifications^2^. For each tested material, 20 samples of standardized dimensions (1.5 mm in thickness and 7.75 mm in diameter) were used. A nylon thread was included in the cement mass of each sample. The initial mass of each specimen was determined using a precision scale (BL 210S, Sartorius AG, Goettingen, Germany). The samples were immersed in 7.5 mL distilled and deionized water. They were connected to the lid of the containers by means of the attached nylon threads, and the assembly was kept in at 37°C for 7 and 30 days. After each period, the samples were removed from the container, washed in distilled water, and placed in a dehumidifier for 24 h. Subsequently, the final mass of each specimen was determined, and the loss of mass was expressed according the percentage of the initial mass.

### Bacterial leakage

Human teeth with a single straight root canal were used. Similar lengths (17 mm) were obtained and the root canals were instrumented up to 1 mm from the apex using MTwo system up to 25.06 instruments under irrigation with 2.5% sodium hypochlorite. The final irrigation was performed with 17% EDTA solution for 3 minutes, followed by irrigation with saline solution. The root canals were dried with paper points.

The root apex was sectioned at 3 mm from the apex. Root end cavities were performed using ultrasonic tip CVD 9.5107-8 and a CVDentus appliance (CVD-Vale, São José dos Campos/SP, Brazil). Retrograde cavities were filled (n=12) with the same materials, manipulated as previously described. Control groups (cavities with no filling and no sealing, n=8; and cavities with no filling and completely sealed, n=8) were also evaluated.

The external surfaces were made impermeable with nail varnish (Colorama, L’Oréal Brazil Commercial Cosmetics Ltda., Rio de Janeiro, RJ, Brazil) and epoxy adhesive (Araldite Fast, Ciba-Geigy AS, Taboão da Serra, SP, Brazil), except 1 mm around the apical surface. Specimens were than mounted to an apparatus with polypropylene microtubes (Eppendorf^®^) and sterilized by ethylene oxide gas (ACECIL, Central de Esterilização Com. Ind. Ltda., Campinas, SP, Brazil). The apical portions of roots were immersed in BHI (Brain Heart Infusion, Difco Laboratories - Becton Dickinson and Company, Franklin Lakes, NJ, USA) and maintained at 37°C for 4 days to confirm its sterility.

A standard strain of *E. faecalis* (ATCC 29212) was used and was cultured in Tryptc Soy Broth – TSB (Difco, Detroit, MI, US) for 24 hours. The optical density of the bacterial suspension was adjusted using a spectrophotometer (Model 600 Plus; Femto, São Paulo, SP, Brazil) to a concentration equivalent to 1x10^9^ CFU mL^-1^. For assessment of bacterial leakage, 500 µL aliquots of standard *E. faecalis* were transferred to the upper portion of the Eppendorf^®^ microtubes contacting the coronal portion of the filling materials. After every seven days (during the experimental period), the BHI inoculated with *E. faecalis* was replaced with a new 500 µL aliquot sterile medium. The removed aliquot was tested to confirm bacterial viability.

Samples were observed daily for 42 days (six weeks), and leakage was detected by turbidity of the BHI medium in contact with the apex of the root. For that, scores from one to seven were established, one for leakage in the first week; two in the second week; and so on, successively. This approach is a modification of the methodology of Oliveira, et al.^21^ (2011).

When the turbidity of the medium was observed, confirmation of cell morphology was carried out by Gram staining. Another portion of turbid BHI was plated on TSA medium to detect growth of E. faecalis and observe colony morphology. The number of leaking samples for each group at different time intervals was observed.

### Statistical analysis

For solubility tests, the data obtained were submitted to parametric and non-parametric ANOVA statistical test, followed by Tukey’s test for multiple comparison (p<0.05). For sealing capacity test, the data obtained were submitted to Kruskal-Wallis test, complemented by Dunn’s test (p<0.05).

## RESULTS

### Solubility

For the 7-day period, ZOE presented highest solubility when compared with the other groups (p<0.05). For the 30-day period, no difference was observed among the groups evaluated (Table 1).

**Table 1:** Mean solubility (% of mass loss) for each period

Test	Period	MTA	CSC/ZrO_2_	ZOE
Solubility	7 days	1.757^a^	1.279^a^	3.157^b^
	30 days	3.471^a^	3.397^a^	4.336^a^

Equal letters in the same column indicate statistical similarity (p>0.05)

### Bacterial leakage

Obtained data are presented in Table 2. According to the average of the scores (MTA: 4.5±1.0; CSC/ZrO_2_: 4.5±0.9; ZOE: 3.1±1.1), a lower level of bacterial leakage was observed for MTA and CSC/ZrO_2_, and both presented better results than ZOE (p<0.05). The positive control presented immediate leakage in all tested samples, whereas the negative control group showed no leakage.

**Table 2:** Number of samples presenting bacterial leakage according to the evaluation weeks

	Number of samples (Scores-week)							Average of the scores
Groups	Week 1	Week 2	Week 3	Week 4	Week 5	Week 6	Week 7	
MTA	0	1	0	4	6	1	0	4.5±1.0^a^
CSC/ZrO_2_	0	0	2	3	6	1	0	4.5±0.9^a^
ZOE	1	2	4	4	1	0	0	3.1±1.1^b^
C+	12	0	0	0	0	0	0	
C-	0	0	0	0	0	0	0	

Equal letters in the same column indicate statistical similarity (p>0.05). C+ = positive control; C- = negative control.

## DISCUSSION

Solubility test, usually analyzed by standardized tests, use the period of 24 hours in water immersion for the analysis of the behavior of the materials. According to this test, the weight loss of each specimen is expressed as the percentage of the original mass (solubility) and the ideal is the value lesser than 3%. The present study evaluates materials solubility after 7-day and 30-day periods. ZOE presented highest solubility in the first period, with a value highest than this 3% (3.157). For the 30-day period, no difference was observed among the evaluated materials. MTA, CSC/ZrO_2_, and ZOE showed values highest than 3% (3.471, 3.397, and 4.336, respectively).

The solubility observed to MTA can be related to bismuth oxide as radiopacifier^6^, which increases its material porosity and changes the longevity of the cement^8^. Analyzing the hydration characteristics of ZrO_2_ associated to Portland cement, Camilleri, et al.^6^ (2011) observed that lower concentration of zirconium was released in solution compared to bismuth in ProRoot MTA, which shows that zirconium oxide is more stable than bismuth oxide in the cement matrix. In the evaluated periods, ZOE solubility may be related to continuous eugenol loss of cement matrix and the leaching effect can justify its values, since the disintegration of the material can induce a mass loss^29^.

Longer experimental periods can be important when analyzing the behavior of the materials. Grech, et al.^14^ (2013) studied fluid solubility until 28 days for a prototype of tricalcium silicate cement, Bioaggregate, Biodentine, and Intermediate Restorative Material (IRM). They observed no difference between the solubility of the materials. In the present study, solubility evaluated after the longer period (30 days) showed no difference between CSC and ZOE. On the other hand, analyzing root canal filling materials after six months using 3D micro-CT analysis, Gandolfi, et al.^12^ (2013) observed that both AH Plus and MTA Flow showed reduction in voids with longer storage times. They suggested that volumetric expansion of the sealers may be responsible for the lower interface porosity detected in the study after six months.

The present results also demonstrated that ZOE and MTA had similar solubility after 30 days. However, bacterial sealing ability was better for MTA and CSC/ZrO_2_. MTA presented only one sample with bacterial leakage on the second week, and this leakage was observed again just on the last weeks, similar to the observed to CSC/ZrO_2._ In contrast, ZOE showed leakage after the first week and lowest bacterial sealing capacity. The higher solubility presented by ZOE after 7 days can be responsible by the earlier bacterial leakage, once the shrinkage of the material can lead to the loss of marginal adaptation. On the other hand, for the 30-day period, no difference was observed among the solubility of the materials. One possible reason for the sealing ability of MTA is its slight expansion after setting^1^ ^,^ ^16^ ^,^ ^28^, which enhances its ability to seal. Another possibility is related to the deposition of crystals in the interface of MTA and calcium silicate cements, increasing the marginal adaptation. Bioactivity is a biological property associated with material ability to release hydroxyl and calcium ions and to develop a stable bond by means of hydroxyapatite deposition^4^ ^,^ ^11^.

The interface of the root filling material and the root canal wall may occur due to the poor adaptation of the material to root dentin, or solubility of the sealer^24^. The bacterial leakage used for this study presents some limitations related to difficulty of standardization^10^ ^,^ ^30^. However, it allows the comparison between the sealing ability of different materials^20^ ^,^ ^22^ ^-^ ^24^. Also, this methodology has clinical relevance, simulating clinical use of root-end filling material^19^ ^,^ ^24^ ^,^ ^26^. Materials were evaluated using similar methodology, and control groups (positive and negative) are performed to show the effectiveness of the method^20^ ^-^ ^22^ ^,^ ^24^. This methodology allows the evaluation of the day of the sample contamination, showed by the broth turbidity, allowing the observation of leakage kinetics during the evaluation period^15^ ^,^ ^24^. The delayed solubility (up to 7 days) for calcium silicate cements may be related to bacterial sealing ability observed for these materials.

The evaluation of the solubility of the materials over longer periods aims to contribute to the understanding of the leakage behavior throughout the study period. Prado, et al.^24^ (2014) evaluated the effect of different irrigant protocols on coronal bacterial leakage of two obturation systems. Samples were contaminated with *Enterococcus faecalis* and monitored every 24 hours for 90 days. Ashraf, et al.^3^ (2013) compared sealing ability of root-end filling material using bacterial leakage model. Teeth were monitored daily for a period of 70 days. In the present study, *Enterococcus faecalis* was used because it invades the dentin tubules and is able to survive in the root canal system, even after root canal treatment. During the experiment, colony morphology and Gram staining confirmed the positive samples**.**

Studies using bacterial leakage have demonstrated MTA sealing capacity^3^ ^,^ ^16^ ^,^ ^25^, corroborating with results of the present study. De-Deus, et al.^9^ (2006) evaluated the bacterial sealing capacity of MTA and Portland cements in furcal perforations for a time interval of 50 days of analysis, showing similar sealing capacity for the materials. The results of the present study demonstrated that the association of CSC/ZrO_2_ did not interfere in the sealing capacity, which was similar to MTA. In this study, a link between solubility and sealing ability was considered. The earlier bacterial leakage of ZOE can be related to a higher solubility in the 7-day evaluation period. Similarly, the less solubility of MTA and CSC/ZrO_2_ in the same period may promote better material adaptation and a later bacterial leakage. Dimensional stability of the material may influence the bacterial leakage. Favorable results of sealing ability for some materials may be related to its dimensional stability, which led to less leakage^7^ ^,^ ^18^. The delayed solubility (up to 7 days) for calcium silicate cements may have favored a longer dimensional stability and bacterial sealing ability observed for these materials.

## CONCLUSIONS

MTA and CSC/ZrO_2_ presented better bacterial sealing capacity, which may be related to less initial solubility observed for these materials in relation to ZOE. Additional studies are needed to establish the material features and correlation between different properties after longer periods.
